# The complete mitochondrial genome of *Anoplocephala perfoliata*, the first representative for the family Anoplocephalidae

**DOI:** 10.1186/s13071-015-1172-z

**Published:** 2015-10-21

**Authors:** Aijiang Guo

**Affiliations:** State Key Laboratory of Veterinary Etiological Biology, Key Laboratory of Veterinary Parasitology of Gansu Province, Lanzhou Veterinary Research Institute, Chinese Academy of Agricultural Sciences, Lanzhou, 730046 Gansu Province People’s Republic of China; Jiangsu Co-innovation Center for Prevention and Control of Important Animal Infectious Diseases and Zoonoses, Yangzhou, 225009 Jiangsu Province People’s Republic of China

**Keywords:** *Anoplocephala perfoliata*, Mitochondrial genome, Phylogenetic analysis

## Abstract

**Background:**

Mitochondrial (mt) genome sequences are widely used to understand phylogenetic relationships among parasites. However, no complete mt genome sequence is available in the family Anoplocephalidae to date. This study sequenced and annotated the complete mt genome of *Anoplocephala perfoliata* (Anoplocephalidae), and investigated its phylogenetic relationships with other species from the families Hymenolepididae, Dipylidiidae and Taeniidae of the order Cyclophyllidea using the amino acid sequences of the 12 proteins in their mt genomes.

**Methods:**

The complete mt genome of *A. perfoliata* was amplified by Long-range PCR, sequenced using primer walking and annotated by comparing with those of other cestodes. Its phylogenetic relationship with the species from the families Hymenolepididae, Dipylidiidae and Taeniidae was inferred using the 12 protein sequences based on Maximum likelihood and Bayesian methods.

**Results:**

The complete circular mt genome sequence for *A. perfoliata* is 14,459 bp in size, and includes 12 protein-coding genes, 2 rRNA genes and 22 tRNA genes. The mt gene arrangement of *A. perfoliata* is identical to those of previously reported *Hymenolepis diminuta* (Hymenolepididae) and *Dipylidium caninum* (Dipylidiidae), but slightly different from those of other taeniids due to an order switch between tRNA(S2) and tRNA(L1). The phylogenetic analyses showed that the Dipylidiidae was more closely related to Anoplocephalidae and Hymenolepididae than to Taeniidae. The relationship among the four families obtained by Maximum likelihood and Bayesian inferences based on predicted amino acid sequences of protein-coding genes is consistent with that based on their mt gene arrangement similarities.

**Conclusions:**

This study determined the first mt genome for the family Anoplocephalidae, providing rich sources for selecting useful molecular markers for ecological and phylogenetic studies. Analyses on mt genome sequences of the four families of cestodes provide novel insights into their phylogenetic relationships. Of couse, more taxon sampling is necessary for future phylogenetic studies of these cestodes using mt genome sequences.

**Electronic supplementary material:**

The online version of this article (doi:10.1186/s13071-015-1172-z) contains supplementary material, which is available to authorized users.

## Background

The Cyclophyllidea order with 15 families is the biggest taxonomic group in Cestoda. The phylogenies of cyclophyllidean tapeworms have been widely studied based on morphological characters [1, 2] which are often convergent and thus of little significance [3, 4]. For example, the apparent similarities are observed between the intermicapsiferine anoplocephalids and the davaineids with fibrous polyovular egg capsules. The Anoplocephalidae is diagnosed in part by the absence of a rostellum when compared with the davaineids. However, the rostellum has been secondarily lost in numerous species or genera within the Davaineidae [1].

Molecular tools, using specific genes and genomic regions (12S rDNA, 28S rDNA, *cox1*, etc.) [3, 5–10], have been used to study phylogenetic relationships among groups of parasitic flatworms. However, some specific genes and genomic regions such as rDNA genes, may not completely provide the useful phylogenetic information at the generic level [6, 11], or may not be the most suitable marker for all taxa [12, 13]. Nevertheless, complete mitochondrial DNA sequences have become effective analytical tools in ecology, evolutionary biology and population genetics [14–18]. The complete mt genomes provide not only individual and combined mtDNA molecular markers for within- and between-species studies but also the information of gene order changes [19]. Platyhelminthe mt genomes comprise 12 protein-coding genes, 2 ribosomal and 22 transfer RNA genes [15], but lack the *atp8* gene. In general, the mitochondrial gene order is rather stable and gene rearrangements are rare evolutionary events. When any common change is discovered within a group, it is considered to be a phylogenetic signal [15, 20, 21]. Although mt genome data provide useful molecular markers for population genetics and phylogenetic studies, there are some families in the cestode order Cyclophyllidea for which mtDNAs are still unavailable to date. Such, few studies on the phylogeny of cyclophyllidean cestodes are conducted at the family level based on complete mitochondrial DNA sequences [11, 14].

Complete mitochondrial genome is unavailable for the Anoplocephalidae family as yet and its systemic relationships with other cestodes in the same order of Cyclophyllidea are currently poorly understood. Several species that belong to the family Anoplocephalidae can cause tapeworm infections in horses and donkeys [22]. Among them, *Anoplocephala perfoliata* is a notable one with a worldwide distribution [23]. In the present study, the complete mitochondrial genome of *A. perfoliata* (Anoplocephalidae) has been sequenced and annotated, which is the first representative of the family Anoplocephalidae. The *A. perfoliata* mt DNA sequence was used along with those of nine published mt genomes of species of cestode families in the Cyclophyllidea, including *Hymenolepis diminuta* (the only complete mt genome available in Hymenolepididae), *Dipylidium caninum* (the only complete mt genome available in Dipylidiidae), *Versteria mustelae*, *Echinococcus granulosus*, *Echinococcus multilocularis*, *Taenia solium*, *Taenia asiatica*, *Hydatigera krepkogorski* and *Hydatigera parva* (seven representative mt genome sequences from the four genera of Taeniidae) to explore family-level phylogenetic relationships of the four families Anoplocephalidae, Taeniidae, Dipylidiidae and Hymenolepididae.

## Methods

### Sampling and DNA extraction

Single *A. perfoliata* adult was collected from the digestive tract of a donkey slaughtered at a commercial abattoir in China for DNA extraction and sequencing. The tapeworm was washed with 0.75 % NaCl. The genomic DNA was extracted using Multisource Genomic Miniprep DNA extraction kit (AXYGEN, USA) following the manufacturer’s instructions.

### Amplification of mtDNA fragments

The complete mitochondrial genomic DNA was amplified by PCR in three overlapping fragments (*nad1*-*rrnS*, *rrnS*-*nad5*, *nad5-nad1*) using three pairs of primers (Additional file 1: Table S4), which were designed according to the published primers [14] used for amplifying short fragments of mtDNAs for all *Taenia* species. PCR reactions were carried out in a 50 μl reaction mixture consisting of 25 μl 2 × buffer (Takara Biotechnology Co. Ltd., Dalian, China), 22.5 μl sterile deionized water, 0.5 μl each primer (50 pmol/μl), and 1.5 μl DNA template (40 ng/μl) in the following conditions: 94 °C for 5 min, then 94 °C for 30 s, 50 °C for 30 s, 68 °C for 8 min for 30 cycles, followed by 68 °C for 10 min. PCR products were purified and underwent sequencing. Each amplicon was sequenced in both directions by primer walking [24] with ABI PRISM 3730 (Applied Biosystems Inc.) at Takara Biotechnology Co. and Shanghai Sangon Co.

### Sequence analyses

The complete mt genomic sequence of *A. perfoliata* was assembled using CAP3 Sequence Assembly Program [25] (http://doua.prabi.fr/software/cap3) and annotated by sequence comparison with those of other cestodes [14, 16] available in the GenBank database. Protein-coding genes were identified using BLAST searches and ORF Finder software at NCBI with the genetic code set for Flatworm (Translation table 9). Transfer RNA (tRNA) genes were annotated using tRNAscan-SE, combining with recognizing anticodon sequences and potential secondary structures by visual inspection. The ribosomal RNA genes (rRNA) were identified by sequence comparisons with the published cestode mt rRNA genes. Palindromes and inverted sequences in the non-coding regions were determined using Einverted and Palindrome in EMBOSS software [26]. Secondary structures were predicted using Mfold software [27]. Tandem repetitive elements were detected with Tandem Repeats Finder program [28]. Nucleotide composition (%) of each gene, non-coding region and the complete mt genome was calculated using DNASTAR’s Lasergene sequence analysis software [29].

### Phylogenetic analyses

A total of 16 mt genome sequences were used for analysis, including seven representative mt genome sequences from the four genera of Taeniidae, and two cyclophyllidean cestode mt genomes available in GenBank (*Hymenolepis diminuta* (Hymenolepididae), and *Dipylidium caninum* (Dipylidiidae)), the mt genome sequence for *A. perfoliata* identified in this study, five mt genomes from Pseudophyiidea (*Spirometra erinaceieuropaei*, *Diphyllobothrium nihonkaiense*, *Kiphyllobothrium latum*, *Diplogonoporus balaenopterae* and *Diplogonoporus grandis*), and mt sequences from the trematode *Schistosoma japonicum* (outgroup) (Additional file 1: Table S1). Phylogenetic analyses were carried out using amino acid sequences, which were predicted from the nucleotide sequences of each protein-coding gene using the flatworm mt genetic code set (Translation table 9). The amino acid sequences for each protein-coding gene of 16 species were aligned individually using MAFFT 7.122 [30]. Poorly aligned positions were removed by Gblocks server (http://molevol.cmima.csic.es/castresana/Gblocks_server.html) [31] using the option for a less stringent selection. A single alignment for phylogenetic analysis was created by concatenating all amino acid alignments of the 12 protein-coding genes. The optimal model of protein evolution was selected by ProtTest [32]. The phylogenetic trees were constructed using Bayesian analysis and Maximum-likelihood (ML). Bayesian analysis was performed by MrBayes 3.2 [33] with mtZoa model (rates = gamma, ngammacat = 5), as suggested by Rota-Stabelli et al. [34]. Two chains (temp = 0.2) were run for 5,000,000 generations and sampled every 1,000 generations. The first 25 % of the trees were treated as burn-in. ML analysis were conducted with PhyML 3.0 [35] using the MtArt + I + G model, and 100 bootstrap replicates were selected to calculated bootstrap support for ML trees.

## Results and discussion

### The complete mt genome organization of *A. perfoliata*

**Fig. 1:**
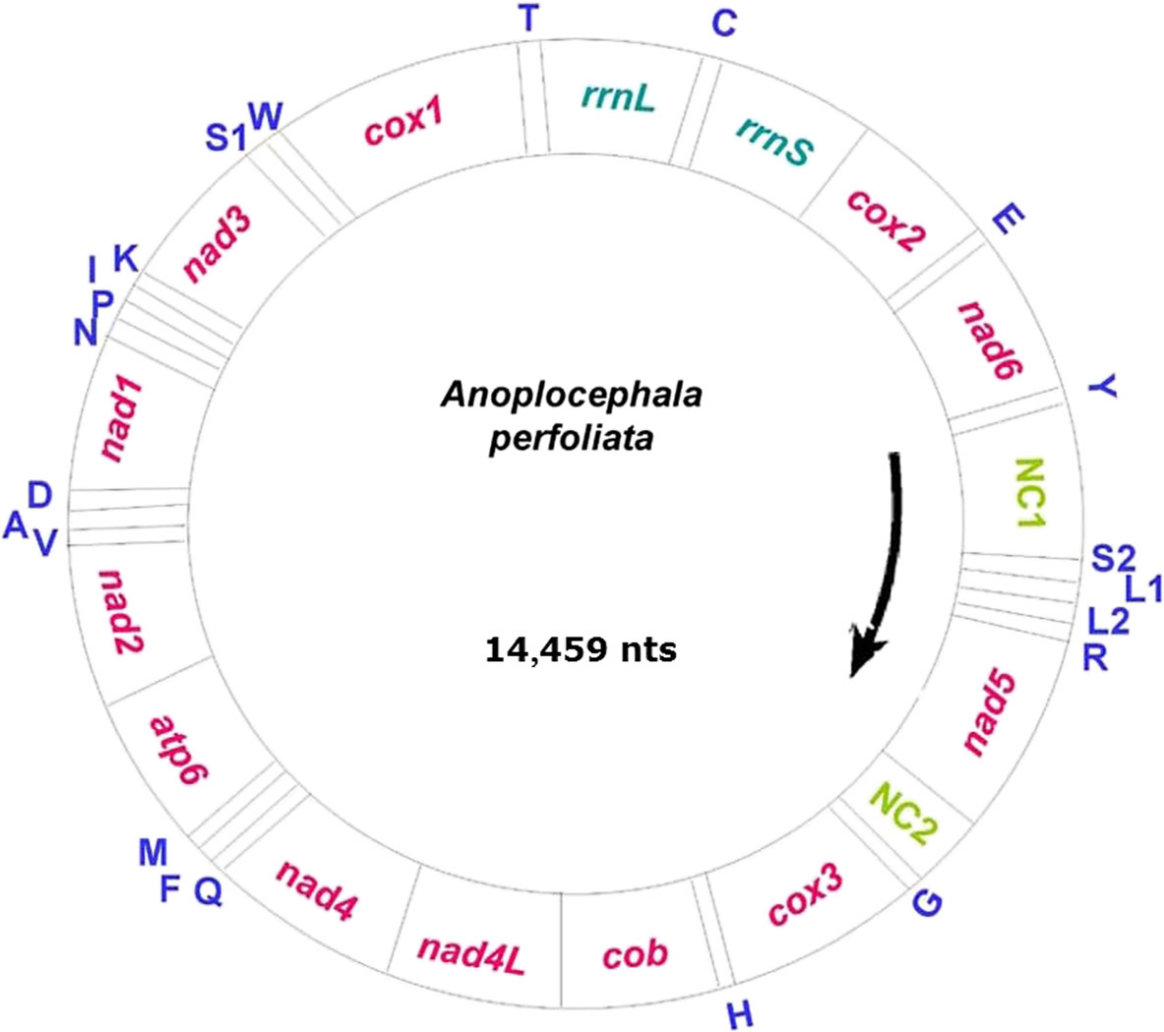
Structure of the mitochondrial genome of *Anoplocephala perfoliata* (also see Table 1). Genome organization of the complete mitochondrial genome of *A. perfoliata* is a circular DNA molecule, which contains 36 genes. All genes are coded by the same DNA strand. Arrow indicates transcriptional orientation. NC refers to non-coding region. 22 tRNA genes are designated with one letter amino acid codes. Gene scaling is approximate

The complete mt genome of *A. perfoliata* (GenBank: KR054960) is a circular, double-stranded DNA molecule with 14,459 bp in size (Fig. 1), which is slightly larger than those of other cestodes (13,482 bp–14,296 bp) [14, 36] (Additional file 1: Table S1). High A + T content (70.99 %) and low G + C content (29.01 %) were observed in the mt genome sequence of *A. perfoliata* (Additional file 1: Table S2). The overall A + T content is very similar to those of other cestode species reported so far (*H. diminuta* [71.04 %], *D. caninum* [72.89 %], *T. solium* [72.1 %], *T. asiatica* [71.4 %], *E. multilocularis* [69.04 %] and *E. granulosus* [67.02 %]). The 12 protein-coding genes (*cox*1-3, *atp*6, *nad*1-6, *nad*4L and *cytb*), 22 tRNA genes and two rRNA genes (*rrnS* and *rrnL*) were identified in *A. perfoliata* mt genome by comparing their sequence identities and/or secondary structures with those of other flatworms. The *atp8* gene that appears in most animal mtDNAs is missing in this *A. perfoliata* isolate, as in other known flatworm mt genomes. All the genes are encoded by one strand in the same direction (Fig. 1). A list of gene arrangement, gene length and intergenic spacer regions of mt genome for *A. perfoliata* are shown in Table 1. It is noteworthy that the gene order of *A. perfoliata* mt genome is identical to those of *H. diminuta* and *D. caninum,* but slightly different from those of other taeniids due to an order switch between tRNA(S2) and tRNA(L1).

**Table 1:** List of annotated mitochondrial genome of *Anoplocephala perfoliata*

Genes	Positions	Lengths (bp)	Initiation and termination codons	Anticodons	Number of aa	Intergenic nucleotides (bp)^a^
*cox*1	1–1593	1593	ATG/TAA	-	530	5
tRNA-Thr (T)	1596–1659	64	-	TGT	-	2
*rrn*L	1660–2640	981	-	-	-	0
tRNA-Cys (C)	2641–2706	66	-	GCA	-	0
*rrn*S	2707–3430	724	-	-	-	0
*cox*2	3431–4006	576	ATG/TAG	-	191	0
tRNA-Glu (E)	4008–4071	64	-	TTC	-	1
*nad*6	4076–4534	459	ATG/TAG	-	152	4
tRNA-Tyr (Y)	4541–4606	66	-	GTA	-	6
Non-coding region (NC1)	4607–5481	875	-	-	-	0
tRNA-SerUCN (S2)	5482–5554	73	-	TGA	-	0
tRNA-LeuCUN (L1)	5582–5642	61	-	TAG	-	27
tRNA-LeuUUR (L2)	5654–5717	64	-	TAA	-	11
tRNA-Arg (R)	5727–5783	57	-	ACG	-	9
*nad*5	5785–7365	1581	ATG/TAG	-	526	1
Non-coding region (NC2)	7366–7644	279	-	-	-	0
tRNA-Gly (G)	7642–7704	63	-	TCC	-	−3
*cox*3	7708–8351	644	ATG/TA	-	214	3
tRNA-His (H)	8352–8418	67	-	GTG	-	0
*cyt*b	8422–9522	1101	GTG/TAG	-	366	3
*nad*4L	9525–9785	261	ATG/TAG	-	86	2
*nad*4	9752–10999	1248	ATG/TAA	-	415	−34
tRNA-Gln (Q)	11001–11063	63	-	TTG	-	1
tRNA-Phe (F)	11062–11123	62	-	GAA	-	−2
tRNA-Met (M)	11120–11184	65	-	CAT	-	−4
*atp*6	11188–11703	516	ATG/TAG	-	171	3
*nad*2	11718–12593	876	ATG/TAG	-	291	14
tRNA-Val (V)	12598–12660	63	-	TAC	-	4
tRNA-Ala (A)	12660–12724	65	-	TGC	-	−1
tRNA-Asp (D)	12726–12789	64	-	GTC	-	1
*nad*1	12793–13683	891	ATG/TAA	-	296	3
tRNA-Asn (N)	13710–13778	69	-	GTT	-	26
tRNA-Pro (P)	13784–13849	66	-	TGG	-	5
tRNA-Ile (I)	13850–13912	63	-	GAT	-	0
tRNA-Lys (K)	13917–13978	62	-	CTT	-	4
*nad*3	13983–14330	348	ATG/TAA	-	115	4
tRNA-SerAGN (S1)	14331–14390	60	-	GCT	-	0
tRNA-Trp (W)	14393–14454	62	-	TCA	-	2

Notes: ^a^shows the length of overlap (−) or intergenic gap (+) between two adjacent genes

### Protein-coding genes

For the 12 protein-coding genes identified in *A. perfoliata*, the *cytb* (1,101 bp) is relatively large in size, whereas *cox1* (1,593 bp) is among the smallest and the remaining ten genes are comparable with the normative sizes, as with the other cestodes mt genomes thus far characterized (Additional file 1: Table S3). Initiation and termination codons inferred from each protein-coding gene are shown in Table 1. Of 12 protein-coding genes, eleven genes were inferred to initiate with the ATG codon except that *cytb* is probably initiated with GTG. Seven were inferred to use TAG as stop codons (*cox2*, *nad6*, *nad5*, *cytb*, *nad4L*, *atp6* and *nad2*), four were inferred to terminate with TAA (*cox1*, *nad4*, *nad1* and *nad3*) and one gene (*cox3*) was terminated with an incomplete stop codon (TA). The truncated incomplete stop codon has also been found in *cox3* gene for *D. caninum*. The initiation and complete or incomplete termination codons for *A. perfoliata* are as normal for flatworm mitochondrial protein-coding genes (Additional file 1: Table S3). Gene overlaps were found between tRNA(F) and tRNA(Q), between tRNA(Q) and tRNA(M), and between tRNA(V) and tRNA(A). The *nad4* gene is inferred to overlap with the upstream *nad4L* gene by 34 nucleotides, which is common for these two genes among metazoan mtDNAs [16].

### Ribosomal RNA and transfer RNA genes

The two ribosomal RNA genes, *rrnS* and *rrnL*, were predicted from the mt genome of *A. perfoliata. rrnS* is located between tRNA(C) and *cox2*, *rrnL* is found between tRNA(T) and tRNA(C), the same position as reported in other cestodes [14, 16]. The length of *rrnL* (992 bp) is relatively larger and *rrnS* (724 bp) is normal, compared to those found in some other cestode species (Additional file 1: Table S3). The A + T contents of *rrnS* and *rrnL* are 69.34 % and 70.67 %, respectively.

**Fig. 2:**
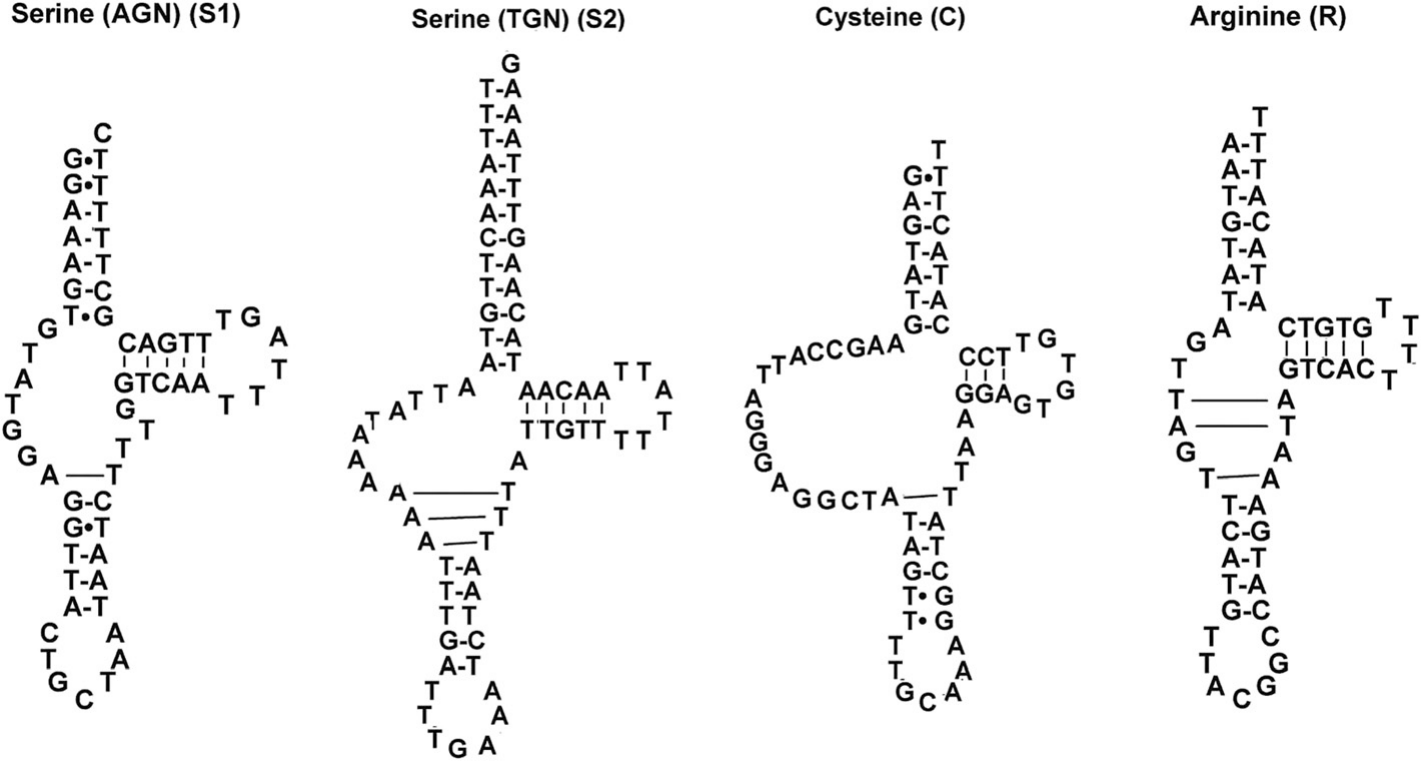
Deduced secondary structures for the four tRNA genes of *Anoplocephala perfoliata* mtDNA are shown. Only these four tRNA genes lack typical cloverleaf structure. Additional possible base-pairing are exhibited with long lines

The 22 tRNA genes were identified in the mtDNA of *A. perfoliata*. The sizes of the tRNA genes are 57–73 bp (Table 1). Predicted secondary structures of 18 tRNAs have the standard cloverleaf structure in *A. perfoliata*, but that of four tRNA genes (tRNA(C), tRNA(R) and two tRNA(S)) lack the DHU arm (Fig. 2), as reported in some *Taenia* species mtDNAs [14]. However, in the tRNAs of the *H. diminuta* mt genome, only three tRNAs (the tRNA(R) and the two tRNA(S) genes) lack the DHU arm. Each of the four tRNAs has the potential for an extra base-pairing, probably allowing a longer anticodon stem in *A. perfoliata* (Fig. 2). Similar tRNA structures with a longer anticodon stem are also found in *Taenia* species [14] and *H. diminuta* mtDNA [16]. Additionally, it is noteworthy that an additional base-pairing for a longer acceptor stem may also be possible for the tRNA(C) in *A. perfoliata* (Fig. 2), yet no similar structure has been reported in other cestode mtDNAs.

### Non-coding sequences

The mt genome of *A. perfoliata* contained two major non-coding regions (NC1 or NC2) (Table 1). NC1 (875 bp) is located between tRNA(Y) and tRNA(S2). NC2 (279 bp) is found between *nad5* and tRNA(G). These are at the same relative positions as in the mt genome of *H. diminuta* [16], but NC1 is larger than NC2 in the *A. perfoliata* mt genome, whereas NC1 is smaller than NC2 in the *H. diminuta* mt genome (Additional file 1: Table S3). These differences in length between the two non-coding regions are less pronounced in cestodes and may not be used as phylogenetic signals [15]. For the other reported taeniid mtDNAs [14], however, the two non-coding regions are placed between tRNA(Y) and tRNA(L1), and between *nad5* and tRNA(G), respectively.

**Fig. 3:**
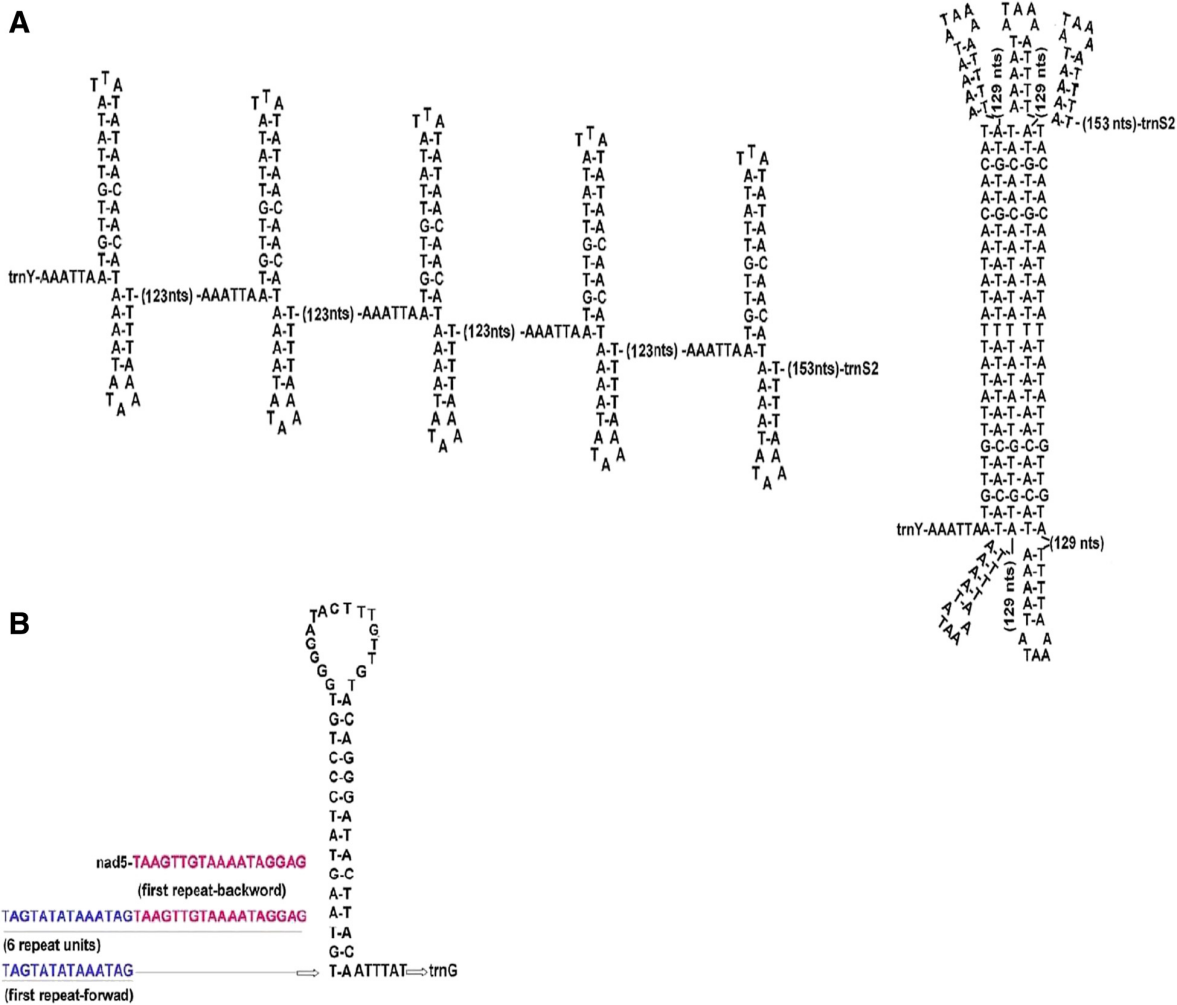
Possible secondary structure for the two non-coding regions in *Anoplocephala perfoliata* mtDNA. **a** Predicted secondary structure in long non-coding region between *trnY* and *trnS2*. Five identical potential secondary structures are shown. Folding shown as pentamer suggests alternative possible hairpins for long non-coding region. **b** Possible secondary structure for the short non-coding region between *nad5* and *trnG.* This structure includes six identical repeat elements and one shuffling repeat element

In the *A. perfoliata* mt genome, NC1 is mainly composed of four repeat elements of 169 bp, plus a part of the repeat element of 85 bp. Only two bases vary among these four repeat elements. The presence of long repeats is a common feature of non-coding regions for cestodes [16]. A number of potential secondary structures in the NC1 of *A. perfoliata* were also predicted. For example, a stable potential secondary structure with two arms and two loops could be folded in each of these repeat regions (Fig. 3a). This secondary structure contains 11 and five canonical base pairs for the stems, with 3 bp and 5 bp loops, respectively (Fig. 3a). For NC1, three two-stem structures are feasible, but a large potential secondary structure presented as a pentamer is also possible with 24 canonical base pairs plus long loops (Fig. 3a). The large potential secondary structures predicted in triplex have been observed for non-coding regions between *nad5* and tRNA(G) or between tRNA(Y) and tRNA(L1) in other cestode mtDNAs [16]. Competitive and mutually exclusive hairpins could be present in this case, as reported in other mtDNAs [16, 37]. Besides the aforementioned major repeat regions, there were two sets of short inverted sequences at the NC1 terminals (TAATACTATTA and ATAAATTTAAAAATTTAT), and each of them could be folded into a hairpin structure.

The second non-coding region (NC2) of the mt genome of *A. perfoliata* comprises six identical repeat elements of a 32 bp nucleotide plus one shuffling repeat element (Fig. 3b). Except for these repeat sequences, the other non-coding sequence with 52 bp in NC2 can form a stem-loop structure with 15 standard base pairs and a loop of 16 bp nucleotides (Fig. 3b). This structure, including identical repeats and a stem-loop in its non-coding region, is only observed in the published mt genome sequences of *H. diminuta* and *Meloidogyne javanica*, which have been predicted for 13 repeat elements with a stem-loop and 11 repeat elements with a stem-loop, respectively [16, 38].

The lengths of non-coding regions in other cestode mt genomes are shown in Additional file 1: Table S3. Similar and stable stem-loop structures can be also found in the non-coding regions of taeniid mtDNAs [14], though the functions of these non-coding regions remain unclear. In mammals, however, similar stable hairpin structures have been shown to initiate replication and transcription [39, 40]. Whether these non-coding regions of cestode mtDNAs serve a similar function remains to be investigated. Other small intergenic regions were discovered between tRNA(S2) and tRNA(L1) (30 bp) and between *nad1* and tRNA(N) (26 bp). Whether any of these non-coding regions serve any function awaits investigation.

### Phylogenetic analyses

**Fig. 4:**
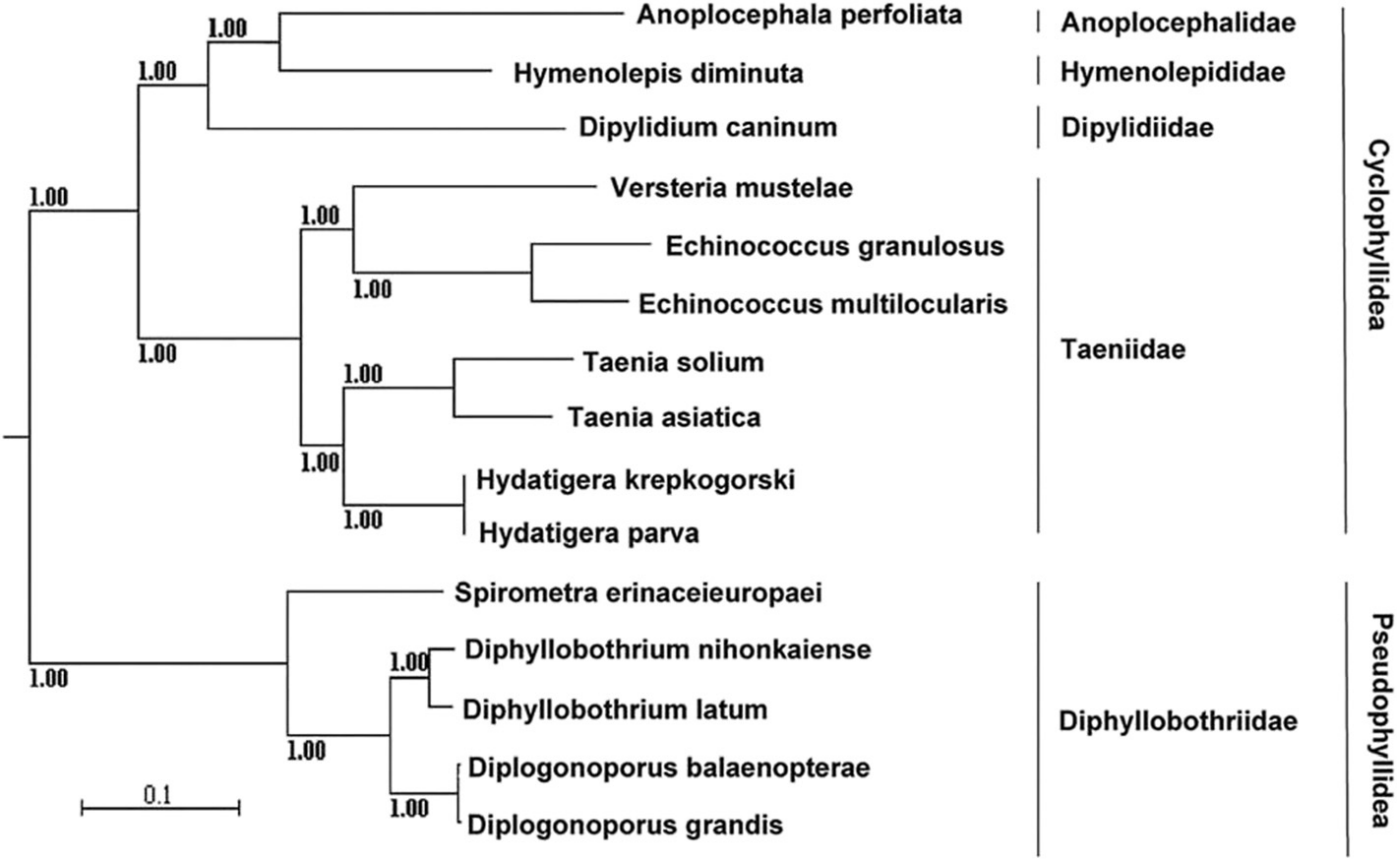
Phylogenetic relationships between Anoplocephalidae, Hymenolepididae, Dipylidiidae and Taeniidae. Phylogenetic tree inferred by the Bayesian inference analysis from deduced amino acids of 12 protein-coding genes is shown. Scale bar indicates number of substitutions per site. Posterior probability values are shown

In order to evaluate the phylogenetic relationship among the families of the order Cyclophyllidea based on mt genome data, phylogeny was reconstructed using inferred amino acid sequences of the 12 protein-coding genes from 16 representatives including ten species from the order Cyclophyllidea, five species from the order Pseudophyllidea and one trematode outgroup. An identical phylogenetic tree topology was obtained through the Maximum-likelihood (ML) and Bayesian analyses (Fig. 4). The tree inferred using the ML method was the same as that of the Bayesian analysis and was omitted. The trees showed that Pseudophyllidea and Cyclophyllidea individuals formed a monophyletic group which is consistent with previous molecular analyses [11, 41, 42]. Within the Taeniidae group, four valid genera were robustly supported with high nodal support. This result is in agreement with the study by Nakao et al. [11], but different from those of other studies suggesting that the cestode family Taeniidae has only two valid genera [11]. The tree revealed sister-group relationship between the Anoplocephalidae and the Hymenolepididae, which agrees with what has been reported using the 18S gene based on distance-based Neighbour-Joining method, but is not consistent with the parsimony-based phylogeny [6]. This inferred relationship between the Anoplocephalidae and the Hymenolepididae is also consistent with some earlier morphological and molecular studies (12S rDNA and *cox1*) suggesting both families as very close parents [3, 5]. Furthermore, the trees indicated that the Dipylidiidae was phylogenetically closer to the Hymenolepididae and the Anoplocephalidae than to the Taeniidae. The evolutionary relationship between these four families revealed in the present study is incongruent with the hypothesis based on the phylogenetic tree derived from 12S rDNA [3] which suggests that the Dipylidiidae is more closely related to the Taeniidae than to either the Hymenolepididae or the Anoplocephalidae, and also disagrees with Hoberg’s proposal [1] that the Hymenolepididae is closer to the Dipylidiidae and the Taeniidae than to the Anoplocephalidae based on comparative morphology analysis.

The present study is the first one to investigate the relationship among these four families using concatenated mt amino acid sequences of 12 protein-coding genes. The relationship among these four families obtained here shows that the Dipylidiidae is more closely related to the Hymenolepididae and the Anoplocephalidae than to the Taeniidae. This can also be supported by the analysis based on similarities of their mt gene arrangement, where the gene arrangement of *A. perfoliata* (Anoplocephalidae) mt genome is identical to those of the species of *H. diminuta* (Hymenolepididae) and *D. caninum* (Dipylidiidae), but different from those of other taeniids. The identical mt gene arrangement among the species of the families Anoplocephalidae, Hymenolepididae and Dipylidiidae supported the result from the Bayesian phylogeny based on concatenated amino acids that these three families might have a closer relationship. Additionally, sequence organization for one of the two non-coding regions in *A. perfoliata* is very similar to that of *H. diminuta*, where the front part of the non-coding region comprises 6 identical repeat elements and the remaining non-coding region sequence can form a stem-loop structure in *A. perfoliata* (Fig. 3b), and 13 repeat elements with a stem-loop structure in *H. diminuta* [16, 38]. Whether the similar feature of the non-coding region between *A. perfoliata* and *H. diminuta* can be used as phylogenetic marker to support the closer relationship between the Anoplocephalidae and the Hymenolepididae is not known yet, a wider sampling will be required to determine. In comparison with other cyclophyllidean tapeworms described earlier [14, 16, 18, 43], mitochondrial genome organization and structure of *A. perfoliata* are more similar with them, supporting the result from the Bayesian phylogeny that the family Anoplocephalidae belongs to the order Cyclophyllidea. Considering the limited mt genome data available for these cestode parasites, additional taxon sampling of the families in the order Cyclophyllidea including the Anoplocephalidae, the Hymenolepididae and the Dipylidiidae is required to fully assess of the inferred phylogenetic relationship.

## Conclusions

In conclusion, this study details the complete sequencing and annotating of the mt genome of *A. perfoliata*. This represents the first complete mtDNA sequence from the family Anoplocephalidae. The complete mtDNA genome contains 36 genes, including 12 protein-coding genes, two ribosomal RNA genes and 22 transfer RNA genes. Phylogenetic analysis inferred from amino acid sequences of 12 protein-coding genes from ten representatives belonging to four families (Hymenolepididae, Dipylidiidae, Taeniidae and Anoplocephalidae) showed that the Dipylidiidae is more closely related to the Hymenolepididae and the Anoplocephalidae than to the Taeniidae. This can also be inferred from the similarities of their mt gene arrangement. This finding provides novel insights into the phylogenetic hypothesis of the four major families of the order Cyclophyllidea. The complete *A. perfoliata* mt genome determined in the present study will provide novel mt genetic markers for studies of the equine tapeworm in epidemiology, molecular identification and diagnosis, and also help to reassess the systematic relationships of cestodes in the order Cyclophyllidea.

## Additional file

Additional file 1:Table S1. Cestode species used for comparative analysis with *Anoplocephala. perfoliata*. **Table S2.** A + T content (%) of the protein-coding, tRNA, rRNA genes and non-coding regions of mitochondrial genome of *Anoplocephala perfoliata*. **Table S3.** Properties of mtDNA protein-coding genes, rRNA genes and non-coding regions of *Anoplocephala perfoliata* and other cestode species. **Table S4.** Primers used to amplify and sequence mitochondrial genome from *Anoplocephala perfoliata.* (DOC 209 kb)
