# Supplementary material for: The complete mitochondrial genome of Anoplocephala perfoliata, the first representative for the family Anoplocephalidae
Source: Parasit Vectors. 2015 Oct 21;8:549. doi: 10.1186/s13071-015-1172-z (PMC4618346; doi:10.1186/s13071-015-1172-z)
Supplement: Additional file 1:Table S1. — Cestode species used for comparative analysis with Anoplocephala. perfoliata. Table S2. A + T content (%) of the protein-coding, tRNA, rRNA genes and non-coding regions of mitochondrial genome of Anoplocephala perfoliata. Table S3. Properties of mtDNA protein-coding genes, rRNA genes and non-coding regions of Anoplocephala perfoliata and other cestode species. Table S4. Primers used to amplify and sequence mitochondrial genome from Anoplocephala perfoliata. (DOC 209 kb) [file 13071_2015_1172_MOESM1_ESM.doc]

**Additional files**

**The complete mitochondrial genome of *Anoplocephala perfoliata*, the first representative for the family Anoplocephalidae**

Aijiang Guo

***Table S1. Cestode species used for comparative analysis with Anoplocephala perfoliata***

| Species | GenBank accession no. | mtDNA Length (bp) |
| --- | --- | --- |
| *Echinococcus multilocularis* | NC_000928 | 13738 |
| *Echinococcus granulosus* | AB786664 | 13610 |
| *Taenia asiatica* | NC_004826 | 13703 |
| *Taenia solium* | NC_004022 | 13709 |
| *Diphyllobothrium nihonkaiense* | EF_420138 | 13607 |
| *Diphyllobothrium latum* | DQ985706 | 13608 |
| *Diplogonoporus grandis* | NC_017615 | 13725 |
| *Diplogonoporus balaenopterae* | NC_017613 | 13724 |
| *Hymenolepis diminuta* | AF314223 | 13900 |
| *Versteria mustelae* | NC_021143 | 13582 |
| *Hydatigera krepkogorski* | NC_021142 | 13792 |
| *Hydatigera parva* | NC_021141 | 13482 |
| *Spirometra erinaceieuropaei* | NC_011037 | 13643 |
| *Dipylidium caninum* | NC_021145 | 14296 |
| *Anoplocephala perfoliata* | KR054960 | 14459 |
| *Schistosoma japonicum* | NC_002544 | 14085 |

**Table S2. A+T content (%) of the protein-coding, tRNA, rRNA genes and non-coding regions of the mitochondrial genome of *Anoplocephala perfoliata*.**

| Regions | T | A | G | C | AT |
| --- | --- | --- | --- | --- | --- |
| Protein-coding | 48.27 | 22.71 | 20.58 | 8.44 | 70.98 |
| *rrn*L | 43.25 | 27.42 | 19.76 | 9.58 | 70.67 |
| *rrn*S | 40.61 | 28.73 | 20.99 | 9.67 | 69.34 |
| tRNAs | 40.82 | 28.94 | 20.09 | 10.15 | 69.76 |
| NC1 | 40.11 | 31.77 | 19.20 | 8.91 | 71.89 |
| NC2 | 32.61 | 42.75 | 22.10 | 2.54 | 75.36 |
| Full genome | 46.05 | 24.95 | 20.48 | 8.53 | 70.99 |

**Table S3. Properties of mtDNA protein-coding genes, rRNA genes and non-coding regions of *Anoplocephala perfoliata* and other cestode species**

| Gene | Species^a^ | | | | | | | | | | | | | | |
| --- | --- | --- | --- | --- | --- | --- | --- | --- | --- | --- | --- | --- | --- | --- | --- |
|  | T. s | T. a | E. g | E.m | H. k | H. p | V.m | D. c | A. p | H. d | D. n | D. l | D. g | D. b | S. e |
| Number of aa | | | | | | | | | | | | | | | |
| Cox3 | 214 | 214 | 215 | 215 | 214 | 214 | 215 | 215 | 214 | 216 | 216 | 216 | 214 | 214 | 214 |
| Cytb | 355 | 355 | 355 | 355 | 355 | 354 | 355 | 360 | 366 | 365 | 368 | 368 | 368 | 368 | 369 |
| Nad4L | 86 | 86 | 86 | 86 | 86 | 86 | 86 | 86 | 86 | 86 | 86 | 86 | 86 | 86 | 86 |
| Nad4 | 403 | 417 | 419 | 404 | 416 | 416 | 419 | 415 | 415 | 409 | 416 | 416 | 416 | 416 | 417 |
| Atp6 | 171 | 171 | 170 | 171 | 172 | 171 | 171 | 171 | 171 | 171 | 169 | 169 | 169 | 169 | 171 |
| Nad2 | 293 | 293 | 293 | 293 | 295 | 294 | 291 | 290 | 291 | 293 | 292 | 292 | 292 | 292 | 290 |
| Nad1 | 297 | 297 | 297 | 297 | 297 | 298 | 297 | 297 | 296 | 296 | 296 | 296 | 296 | 296 | 296 |
| Nad3 | 115 | 115 | 115 | 115 | 113 | 113 | 115 | 114 | 115 | 115 | 118 | 118 | 118 | 118 | 115 |
| Cox1 | 539 | 539 | 557 | 535 | 543 | 538 | 539 | 580 | 530 | 517 | 521 | 521 | 521 | 521 | 521 |
| Cox2 | 193 | 191 | 193 | 193 | 194 | 194 | 192 | 191 | 191 | 192 | 189 | 189 | 189 | 189 | 189 |
| Nad6 | 150 | 150 | 151 | 151 | 150 | 149 | 152 | 151 | 152 | 152 | 152 | 152 | 152 | 152 | 155 |
| Nad5 | 522 | 522 | 523 | 524 | 522 | 524 | 522 | 521 | 526 | 524 | 525 | 522 | 522 | 522 | 522 |
| Length of rRNA gene (bp) | | | | | | | | | | | | | | | |
| rrnL | 980 | 975 | 967 | 983 | 960 | 961 | 963 | 970 | 992 | 967 | 964 | 968 | 963 | 962 | 973 |
| rrns | 705 | 731 | 726 | 704 | 728 | 730 | 727 | 729 | 724 | 709 | 743 | 740 | 731 | 730 | 730 |
| Length of non-coding region (bp) | | | | | | | | | | | | | | | |
| NC1 | 68 | 70 | 66 | 183 | 175 | 95 | 73 | 702 | 875 | 183 | 224 | 222 | 226 | 125 | 204 |
| NC2 | 192 | 176 | 184 | 177 | 397 | 194 | 197 | 212 | 279 | 443 | 331 | 188 | 320 | 321 | 174 |
| Deduced initiation codon | | | | | | | | | | | | | | | |
| Cox3 | ATG | ATG | ATG | ATG | ATG | ATG | ATG | ATG | ATG | ATG | GTG | GTG | GTG | GTG | GTG |
| Cytb | ATG | ATG | ATG | ATG | ATG | ATG | ATG | ATG | GTG | ATG | ATG | ATG | ATG | ATG | ATG |
| Nad4L | ATG | ATG | GTG | GTG | ATG | ATG | ATG | ATG | ATG | ATG | ATG | ATG | ATG | ATG | ATG |
| Nad4 | GTG | ATG | ATG | ATG | GTG | ATG | ATG | GTG | ATG | ATT | ATG | ATG | ATG | ATG | ATG |
| Atp6 | ATG | ATG | ATG | ATG | ATG | ATG | ATG | ATG | ATG | ATG | ATG | ATG | ATG | ATG | ATG |
| Nad2 | ATG | ATG | ATG | ATG | ATG | ATG | ATG | ATG | ATG | ATG | ATG | ATG | ATG | ATG | ATG |
| Nad1 | ATG | ATG | GTG | ATG | GTG | ATG | ATG | ATG | ATG | ATG | ATG | ATG | ATG | ATG | ATG |
| Nad3 | ATG | ATG | ATG | ATG | ATG | ATG | ATG | ATG | ATG | ATG | ATG | ATG | ATG | ATG | ATG |
| Cox1 | ATG | ATG | ATG | ATG | ATG | ATG | ATG | ATG | ATG | TTT | ATG | ATG | ATG | ATG | ATG |
| Cox2 | ATG | ATG | GTG | GTG | ATG | ATG | ATG | ATG | ATG | ATG | ATG | ATG | ATG | ATG | ATG |
| Nad6 | ATG | GTG | ATG | ATG | GTG | ATG | ATG | ATG | ATG | ATG | ATG | ATG | ATG | ATG | ATG |
| Nad5 | ATG | ATG | ATG | ATG | ATG | ATG | ATG | ATG | ATG | ATG | ATG | ATG | ATG | ATG | ATG |
| Deduced termination codon | | | | | | | | | | | | | | | |
| Cox3 | TAG | TAG | TAG | TAG | TAA | TAA | TAG | T | TA | TAG | TAG | TAG | T | T | T |
| Cytb | TAA | TAA | TAA | TAA | TAA | TAA | TAA | TAG | TAG | TAG | TAA | TAA | TAG | TAG | TAA |
| Nad4L | TAA | TAG | TAA | TAG | TAG | TAA | TAG | TAG | TAG | TAG | TAA | TAA | TAG | TAG | TAG |
| Nad4 | TAG | TAG | TAG | TAG | TAG | TAA | TAA | TAA | TAA | TAG | TAG | TAG | TAG | TAG | TAG |
| Atp6 | TAA | TAA | TAG | TAG | TAG | TAG | TAA | TAG | TAG | TAG | TAG | TAG | TAA | TAA | TAA |
| Nad2 | TAA | TAA | TAG | TAG | TAA | TAA | TAG | T | TAG | TAG | TAG | TAG | TAA | TAA | TAG |
| Nad1 | T | T | TAA | TAG | TAG | TAG | TAG | TAA | TAA | TAG | TAG | TAG | TAG | TAG | TAA |
| Nad3 | TAG | TAG | TAG | TAA | T | TAG | TAG | TAG | TAA | TAG | TAG | TAG | TAG | TAG | T |
| Cox1 | TAG | TAA | TAA | TAG | TAA | TAG | TAG | TAA | TAA | T | TAG | TAG | TAG | TAG | TAG |
| Cox2 | TAG | TAG | TAG | TAG | T | T | TAG | TAG | TAG | TAA | TAA | TAA | TAA | TAA | TAA |
| Nad6 | TAG | TAA | TAG | TAA | TAG | TAA | TAA | TAG | TAG | TAA | TAG | TAG | TAA | TAA | TAA |
| Nad5 | TAA | TAA | TAA | TAA | TAG | TAA | TAA | TAA | TAG | TAG | TAA | TAA | TAA | TAA | TAA |

Note: Results obtained from GenBank accessions.

^a^Abbreviations: T.s: *Taenia solium*; T.a: *Taenia asiatica*; E.g: *Echinococcus granulosus*; E.m: *Echinococcus multilocularis*; H.k: *Hydatigera krepkogorski*; H.p: *Hydatigera parva*; V.m: *Versteria mustelae*; D.c: *Dipylidium caninum*; A.p: *Anoplocephala perfoliata*; H.d: *Hymenolepis diminuta*; D.n: *Diphyllobothrium nihonkaiense*; D.l: *Diphyllobothrium latum*; D.g: *Diplogonoporus grandis*; D.b: *Diplogonoporus balaenopterae*; S.e: *Spirometra erinaceieuropaei*

***Table S4. Primers used to amplify and sequence the mitochondrial genome from A. perfoliata***

| Primer (heavy strand) | Position based on *A. perfoliata* mtDNA | Sequence (5’ to 3’) |
| --- | --- | --- |
| AP1F_ND1 | 12892-12917 | CARTTTCGTAAGGGBCCWAAWAAGGT |
| AP1R_rrnS | 3362-3396 | AATTCATTTAAAGTTACCTTGTTACGACTTACCTC |
| AP2F_rrnS | 2850-2880 | AGGGGATAGGRCACAGTGCCAGCATCTGCGG |
| AP2R_ND5 | 6302-6323 | GGAAAHCTAGCACTCTTDGTAA |
| AP3F_ND5 | 5670-5700 | TATATGAGTTAGTTTTAAGCATTAATTATGG |
| AP3R_ND1 | 13736-13752 | CCATTTCYTGAAGTTAACAGCATCA |
| F1 | 234-257 | AGGTGGGTTTGGAAATTATTTGTT |
| F2 | 738-759 | AATTTTGCCTGGGTTTGGGATT |
| F3 | 2236-2258 | GGCTTGTTTGAATGGTTTGATGT |
| F4 | 2847-2868 | TAAAGGGGATAGGACACAGTGC |
| F5 | 3359-3380 | GTTGAGGTAAGTCGTAACAAGG |
| F6 | 3565-3587 | TCAGGTAGTGGAGTTAATGTGGA |
| F7 | 4579-4603 | TTTGAAGGTGAAATTTTCCTGATCA |
| F8 | 5989-6010 | ACAGGGCATTATTTTGGTGGAG |
| F9 | 6519-6543 | TGTGGATTTAAAGAAGATTGTTGCT |
| F10 | 7167-7191 | TGTTGAGGGATTTTATGAGTTGTTT |
| F11 | 8034-8056 | TCATCATCTTTTAGGGTGGAAGT |
| F12 | 9182-9206 | TGAGCACTCCTATTAGTATTAAGCC |
| F13 | 10467-10488 | TTTTCTTTATCACCGCGGCTAG |
| F14 | 11464-11486 | TGTCCTTTAGTATGTTTGGCAGA |
| F15 | 12083-12107 | TTTGAGAGTGATTTTGAAGTTTCCT |
| F16 | 12809-12830 | TTTCTGGTGGTTTGGGCTTATT |
| F17 | 13294-13316 | TTTTATTGGGGAAGATGCGTTTT |
| F18 | 13503-13525 | GGTTGCAGTTATTATATGGGGG |
| F19 | 13750-13773 | TCAAGAAGAGGTGATTAACTCTGT |
| R1 | 366-387 | AAAAGTTCAACCTACCCCAGCA |
| R2 | 609-630 | TAAAAGCATCGTTATTGCCGCA |
| R3 | 1111-1132 | AATGCCCAACAACAAATCAAGT |
| R4 | 2096-2117 | CAGACGGACAAACTTCGAGAAA |
| R5 | 2612-2633 | AAGCTTATGGTCCTTTCGTACT |
| R6 | 2847-2868 | GCACTGTGTCCTATCCCCTTTA |
| R7 | 3936-3957 | ACTCCACATAACTCAGCACAAT |
| R8 | 5458-5479 | CAAAACGGTTACTCCTTCCTCA |
| R9 | 5700-5721 | ATCGAATAGCTAGGATTTCCTC |
| R10 | 6391-6414 | TCAAATTCCTGCAGCTACTAAAGT |
| R11 | 7167-7191 | AAACAACTCATAAAATCCCTCAACA |
| R12 | 8890-8911 | CCTCTGTAACACTAAAGCCACC |
| R13 | 9242-9263 | CCTAGCTTAGACTCCACCCATC |
| R14 | 11830-11851 | ATAGAAAGACCACCAAGCTCCA |
| R15 | 12158-12182 | CGCTATAGTAAGAAAACAATCCCCA |
| R16 | 12418-12439 | GGGAAAGAAAAAGGAGTAACCA |
| R17 | 13142-13163 | CCTCAACCAGCACAGAGTAAAG |
| R18 | 14093-14114 | AAACCCACATTCATAAGGCCTC |
